# Physical Activity Is Linked to Greater Moment-To-Moment Variability in Spontaneous Brain Activity in Older Adults

**DOI:** 10.1371/journal.pone.0134819

**Published:** 2015-08-05

**Authors:** Agnieszka Z. Burzynska, Chelsea N. Wong, Michelle W. Voss, Gillian E. Cooke, Neha P. Gothe, Jason Fanning, Edward McAuley, Arthur F. Kramer

**Affiliations:** 1 The Beckman Institute for Advanced Science and Technology at the University of Illinois, Urbana, IL, United States of America; 2 Department of Psychological and Brain Sciences, University of Iowa, Iowa City, IA, United States of America; 3 Department of Kinesiology, Health and Sport Studies, Wayne State University, Detroit, MI, United States of America; 4 Department of Kinesiology and Community Health, University of Illinois, Urbana, IL, United States of America; University of Western Ontario, CANADA

## Abstract

Higher cardiorespiratory fitness (CRF) and physical activity (PA) in old age are associated with greater brain structural and functional integrity, and higher cognitive functioning. However, it is not known how different aspects of lifestyle such as sedentariness, light PA (LI-PA), or moderate-to-vigorous physical activity (MV-PA) relate to neural activity in aging. In addition, it is not known whether the effects of PA on brain function differ or overlap with those of CRF. Here, we objectively measured CRF as oxygen consumption during a maximal exercise test and measured PA with an accelerometer worn for 7 days in 100 healthy but low active older adults (aged 60–80 years). We modeled the relationships between CRF, PA, and brain functional integrity using multivariate partial least squares analysis. As an index of functional brain integrity we used spontaneous moment-to-moment variability in the blood oxygenation level-dependent signal (SD_BOLD_), known to be associated with better cognitive functioning in aging. We found that older adults who engaged more in LI-PA and MV-PA had greater SD_BOLD_ in brain regions that play a role in integrating segregated functional domains in the brain and benefit from greater CRF or PA, such as precuneus, hippocampus, medial and lateral prefrontal, and temporal cortices. Our results suggest that engaging in higher intensity PA may have protective effects on neural processing in aging. Finally, we demonstrated that older adults with greater overall WM microstructure were those showing more LI-PA and MV-PA and greater SD_BOLD_. We conclude that SD_BOLD_ is a promising correlate of functional brain health in aging. Future analyses will evaluate whether SD_BOLD_ is modifiable with interventions aimed to increase PA and CRF in older adults.

## Introduction

Higher cardiorespiratory fitness (CRF) and physical activity (PA) in old age is associated with greater brain structural and functional integrity, and higher cognitive functioning [1–3]. In this study we extend our understanding of the different and overlapping roles of CRF and PA in brain resting state function in healthy but low-active older adults.

There are three main challenges in understanding how physical health and lifestyle PA relate to brain function in older adults. First, it is not known how non-exercise lifestyle activities, such sedentariness (prolonged and uninterrupted periods of sitting, such as watching TV) or light PA (LI-PA; housework, gardening, relaxed walking) relate to brain function, although they account for the majority of daily waking time. This is because lower intensity PA, in contrast to exercise-related moderate-to-vigorous PA (MV-PA, e.g. jogging, walking stairs, biking), is not well captured by self-reports [4]. This is important, as sedentariness and MV-PA are associated with different physiological mechanisms and may differentially affect brain health. For example, general sedentariness may negate or weaken the benefits of sporadic MV-PA: it is possible to be an “active couch potato” who has normal CRF due to bouts of exercise, but is not immune to glucose and fat metabolism risk caused by prolonged sitting [5–7]. Second, it is not known how the effects of CRF and PA differ or overlap with respect to brain function. CRF, measured as maximal oxygen consumption during maximal physical effort, is a sum of various factors, such as pulmonary diffusion capacity, cardiac output, erythrocyte levels, muscle capillary density and respiration rate [8–10], many of which are genetically determined, at least in part [11]. Therefore, although CRF and PA tend to be related, they may not be equivalent in their relationships to brain health. Indeed, our recent work indicates that PA is more predictive of white matter (WM) microstructure than CRF, and different levels of PA intensity relate to different aspects of WM health. Specifically, less time spent sitting was related to microstructure of WM near the hippocampus, more LI-PA was related to temporal lobe microstructure, while older adults spending more time in MV-PA had lower volume of WM lesions [12]. Still, these objective measures of time spent in different intensities of PA have not yet been related to functional brain health in aging. To address these two challenges, we objectively measured PA with an accelerometer, which was worn for 7 consecutive days during all waking hours, in addition to CRF.

The third challenge is measuring brain function and the interpretation of the fitness- and PA-brain function relationships. For example, depending on brain region and task, greater CRF is associated with either increased or decreased change in blood oxygenation level dependent (BOLD) signal, a proxy for neural activity [13–15]. As a result, it is unclear whether high or low amplitudes of BOLD signal reflect optimal functional brain health, and how this association varies regionally throughout the brain. Similarly, a moderate intensity walking intervention was related to increased functional connectivity within the default mode network [16], while the association between CRF and functional connectivity in middle-aged adults was both positive and negative, depending on whether local or long-distance connections were considered [17]. To overcome the challenges in interpretation of conventional positive/negative task-related changes in BOLD signal or different scales of functional synchrony between distinct brain regions, here we employed a more general measure of neural function: moment-to-moment variability in the BOLD signal during spontaneous brain activity.

Moment-to-moment variability in the BOLD signal (SD_BOLD_) is known to reflect the dynamic range of neural processing, such as the modulation of functional networks and is suggested to be a promising tool in mapping neural correlates of cognitive abilities in aging [18,19]. Specifically, lower SD_BOLD_ in certain brain regions is associated with older age, slower, and less consistent performance on a perceptual matching task [20], as well as lower performance on memory and reasoning tasks [21]. These findings suggest there is some optimal range of SD_BOLD_ that may be decreasing with age in certain brain regions, and that older adults differ in their deviation below this optimum, which is associated with individual differences in cognitive performance.

In this study, we sought to determine how the level of physical fitness (measured as CRF) and PA (measured via accelerometer) are related to functional brain health measured as SD_BOLD_. To this end, we collected resting functional magnetic resonance BOLD data from 100 healthy older participants (60–80 years). We modeled the relations between whole-brain voxel-wise SD_BOLD_, CRF, and PA (mean daily sedentary time, time spent in LI-PA, and MV-PA) within a multivariate partial least squares framework [22]. Given that: 1) advancing age is associated with decreasing SD_BOLD;_ and 2) greater CRF, PA, and lower sedentariness are associated with better cognitive and brain health outcomes in older adults, we predicted that greater SD_BOLD_ in certain regions would reflect greater brain health and therefore positively correlate with CRF and PA, and negatively with sedentariness. Although the relevance of SD_BOLD_ in the brain for physical fitness is not yet known, we hypothesized that some of the regions where conventional measures of structural and functional brain integrity have been previously associated with greater CRF (e.g. hippocampus, temporal lobe, core regions of the default mode network (precuneus and medial prefrontal cortex), fronto-parietal regions of the central executive network, anterior cingulate, insula, and thalamus) would show this CRF/PA—SD_BOLD_ relationship [13–16,23–25].

In sum, we have shown previously that more active and less sedentary older people have greater WM microstructural integrity and lower volume of WM lesions [12], that WM microstructure is positively related to behaviorally relevant SD_BOLD_ [21], and that overall WM microstructure provides a scaffold for neural processing in the grey matter (GM) [26]. Therefore, we tested the hypothesis that WM microstructural integrity should be positively associated with CRF/PA-related SD_BOLD_. To this end, we used diffusion tensor imaging (DTI) to infer about WM microstructure by quantifying the magnitude and directionality of diffusion of water within a tissue. We used fractional anisotropy (FA) averaged over all major WM tracts as a summary measure of fiber density, coherence, and myelination degree [27–29].

We found that older adults who spend more time daily on LI-PA and MV-PA had greater SD_BOLD_ in multiple brain regions, and this relationship was positively associated with WM microstructure.

## Methods

### Participants

A University of Illinois Institutional Review Board approved the study, and written informed consent was obtained from all participants and the study was performed in accordance with the 1964 Declaration of Helsinki. Participants received financial reimbursement.

We collected MRI, PA and CRF data from 150 community-dwelling healthy older adults (51 males). The sample contained more females because fewer older males responded to recruitment materials and met the following inclusion criteria: (1) were between the ages of 60 and 80 years old; (2) were free from psychiatric and neurological illness and had no history of stroke or transient ischemic attack; (3) scored ≥ 23 on the Mini-Mental State Exam (MMSE [30]) and >21 on a Modified Telephone Interview of Cognitive Status (TICS-M [31]) questionnaire; (4) scored < 10 on the geriatric depression scale (GDS-15 [32]);, (5) scored ≥ 75% right-handedness on the Edinburgh Handedness Inventory [33];, (6) demonstrated normal or corrected-to-normal vision of at least 20/40 and no color blindness;, (7) were cleared for suitability in the MRI environment, that is, no metallic implants that could interfere with the magnetic field or cause injury, no claustrophobia, and no history of head trauma. The participants were a pre-intervention cross-sectional subsample from an on-going randomized controlled exercise trial (“Influence of Fitness on Brain and Cognition II” at ClinicalTrials.gov, clinical study identifier NCT01472744). We further excluded participants with MMSE < 27 to limit the analyses to cognitively healthy older adults and exclude those with possible mild cognitive impairment. This resulted in a sample of 133 participants (45 male). Out of 133, 100 participants (34 males) had good quality MRI data available (see section on resting state and MPRAGE) and only these datasets were considered for further analyses (age range 60–78, M_age_ = 65.4 ± 4.4 years, years of education 12–26, M_edu_ = 16.8 ± 3.5 years).

### Physical activity assessment

Participants were instructed to wear the GT3X ActiGraph accelerometer (ActiGraph; Pensacola, Florida) for 7 consecutive days on an elastic belt on the left (non-dominant) hip during all waking hours, except for when bathing or swimming. The participants completed a daily log to record the time that the accelerometer was worn, and this log was used to verify the accelerometer data for processing with the ActiLife v5.6.0 software. For the purposes of this study, a valid day of data consisted of at least 10 hours of valid wear-time, with a valid hour defined as no more than 30 consecutive minutes of zero counts with one minute sampling epochs. Only data for individuals with a minimum of 3 valid days of wear time were included in analyses [34]. The 100 participants had on average 6.8 ± 0.8 valid days of measurement (range 4–8), resulting in 95% of the sample having 6 or more valid days required to reliably measure sedentary behavior [34].

Each valid measurement epoch (minute) was classified into sedentary, light, moderate, and vigorous physical activity based on displacement magnitude and frequency. We used activity intensity cut-off ranges appropriate for older adults [35] using MeterPlus v4.2 software (Santech, Inc.; San Diego, CA). Sedentary behavior was defined as <100, light activity as 100–1951, moderate activity as 1952–5723, and vigorous activity as > 5724 counts/minute [35]. The total minutes of each intensity, divided by total valid days, yielded average time (in hours or minutes) spent daily in a specific physical activity intensity (Table 1). Only 15 out of 100 participants showed any vigorous activity during the measurement week. We therefore summed moderate and vigorous activity to obtain a “moderate-to-vigorous activity” (MV-PA) variable [36]. Observed MV-PA was positively skewed and we performed a natural log-transformation of this variable for further analyses.

**Table 1:** Descriptive statistics and correlations with age.

Variable	n	Mean±SD	Range	*r* with age	p-value
*CRF* (VO_2_max ml/kg/min)					
Males	34	26±9	11–47	**-.44**	.**010**
Females	66	20±6	9–39	-.17	.168
*Physical activity*					
*(hours or min/day)*					
Sedentary	100	8.9±1.2hrs	5.8–11.6	.10	.311
LI-PA	100	4.6±1.2hrs	2.3–8.7	-.06	.548
MV-PA	100	17±17min*	0.83min–1.28hour*	**-.32** **	.**001**
*Fractional anisotropy*	71	.46±.02	39–.51	**-.36**	**.002**

*Raw data.

** MV-PA were ln-transformed for correlations with age. There were gender differences only for CRF and not for any other variables.

The sample was defined as low-active and low-fit but generally healthy and living independently, because the participants had to be eligible for the subsequent exercise intervention study (capable of performing exercise, i.e. no physical disability that prohibits mobility) and were expected to benefit from such lifestyle change. To define our sample we used both the self-reports at study entry and verified them with the subsequent objective accelerometry data. Eighty participants at study entry reported not to have participated in regular PA (maximum of two moderate bouts of PA/week) in the past six months. The remaining 20 reported engaging in some exercise upon recruitment. However, the subsequent accelerometer data analysis revealed that only 32 of the total sample (17 from the 80 self-reported low-active individuals and 15 of the 20 more active participants) met the minimum recommendations for PA (>150 min of moderate PA per week; [37]). This means that roughly ¼ of participants in each subgroup (active or not active by self-report) incorrectly assessed their PA levels. Clearly, this discrepancy between self-reports and accelerometer data highlights the necessity of objective assessment of PA for accurate sample description when studying the relationships between brain health and PA in older adults. Importantly, the accelerometer data reflects all PA within the 7-day period, while the PA recommendations refer to leisure time or planned exercise PA, in addition to the lifestyle-related PA. Therefore, accelerometer estimates of PA include both lifestyle activities (shopping, climbing stairs) and leisure exercise (jogging, bike trips). As follows, even less than 32 participants met the actual PA recommendations and, therefore, we defined our sample as low-fit and low-active.

### Cardiorespiratory fitness assessment

All participants obtained physician's approval to engage in cardiorespiratory fitness (CRF) testing. CRF was defined as peak oxygen consumption [ml/kg/min], measured with indirect calorimetry during a modified Balke graded maximal exercise test on a motor-driven treadmill test. Oxygen consumption (VO_2_) was calculated from expired air sampled at 30-s intervals until peak VO_2_ was reached or the test was terminated due to volitional exhaustion and/or symptom limitation. CRF was defined as the highest recorded VO_2_ value (VO_2_max) after two of three criteria were met: (1) a plateau in VO_2_ after increase in workload; (2) a respiratory exchange ratio >1.10; and (3) a maximal heart rate within 10bpm of their age-predicted maximum. Our subjects represented a broad range of CRF values that were normally distributed and fell within the 90% peak VO_2_max percentile (very poor to good, Table 1) according to gender- and age-specific norms (ACSM's Guidelines for Exercise Testing and Prescription, www.acsm.org). As men and women differ in VO_2_max due to body composition, lung size etc., we transformed VO_2_max values into z-scores within each gender group to remove variance related to sex.

### MRI acquisition

We acquired all images during a single session on a 3T Siemens Tim Trio system with 45 mT/m gradients and 200 T/m/sec slew rates (Siemens, Erlangen, Germany). T2*-weighted resting state images were acquired with fast echo-planar imaging (EPI) sequence with Blood Oxygenation Level Dependent (BOLD) contrast (6min, TR = 2s, TE = 25ms, flip angle = 80 degrees, 3.4 x 3.4 mm^2^ in-plane resolution, 35 4mm-thick slices acquired in ascending order, Grappa acceleration factor = 2, 64 × 64 matrix), while the participants were asked to lie still with eyes closed. Additionally, gradient field maps were acquired to account for geometric distortions caused by magnetic field inhomogeneity [38]. The gradient field map was collected as 35, 4mm-thick slices, 3.4 x 3.4 mm^2^ in-plane resolution, TR = 700ms, TE = 10ms, and flip angle = 35 degrees. DTI images were acquired with a twice-refocused spin echo single-shot Echo Planar Imaging sequence [39] to minimize eddy current-induced image distortions. The protocol consisted of a set of 30 non-collinear diffusion-weighted acquisitions with b-value = 1000s/mm^2^ and two T2-weighted b-value = 0 s/mm^2^ acquisitions, repeated two times (TR/TE = 5500/98 ms, 128 x 128 matrix, 1.7x1.7 mm^2^ in-plane resolution, FA = 90, GRAPPA acceleration factor 2, and bandwidth of 1698 Hz/Px, comprising 40 3-mm-thick slices). Resting state, fieldmap, and diffusion images were obtained parallel to the anterior-posterior commissure plane with no interslice gap.

High-resolution structural MR scans were acquired using a 3D MPRAGE T1-weighted sequence (TR = 1900 ms; TE = 2.32 ms; TI: 900 ms; flip angle = 9°; matrix = 256 × 256; FOV = 230mm; 192 slices; resolution = 0.9 × 0.9 × 0.9 mm; GRAPPA acceleration factor 2) and used as an intermediate step in registration of functional images to standard MNI space.

### BOLD variability (SD_BOLD_) calculation

Data processing was carried out using FSL v5.0.1 (FMRIB's Software Library, http://www.fmrib.ox.ac.uk/fsl; Smith et al. 2004). The preprocessing included filtering out frequencies <0.008Hz, slice timing correction, rigid body motion correction using MCFLIRT [40], and removal of non-brain tissue with the Brain Extraction Tool [41]. Data were screened for motion and all participants moved within a voxel dimension (< 4mm). Functional images of each participant were aligned to the standard stereotaxic space of the MNI 152 T1 2mm^3^ template supplied in FSL in a three-step procedure. To improve the registration between the participant’s functional and anatomical images we utilized the gradient field map data. First, the gradient field map was unwrapped via PRELUDE [42], then geometric distortions in the EPI-related images due to local magnetic inhomogeneity differences were compensated for with the use of gradient field map data via FUGUE within FSL [43]. Ten out of 100 participants had missing field map images. Second, each participant’s low-resolution functional images were aligned with their high-resolution T1-weighted anatomical images using the Boundary-Based Registration in FSL [44]. Third, the anatomical images were aligned to MNI 152T1 2mm^3^ template using 12 degrees of freedom affine linear registration [40].

Next, we used Multivariate Exploratory Linear Optimized Decomposition into Independent Components (MELODIC v3.10) tool in FSL [45] to decompose the 4D fMRI time series into spatial and temporal components using independent component analysis (ICA). AZB together with Chanheng He and CNW identified artifact components for each subject using the criteria outlined in [46] based on the spatial pattern, time course, and power spectrum properties that were characteristic of physiological noise, motion, and scanner-related artifacts. We note here that motion timecourses were viewed simultaneously with the components’ timecourses, which made it straightforward to classify motion components as noise. The artifactual components were regressed out from the time series yielding the post-ICA ‘cleaned’ data. These post-ICA functional data were further low-pass filtered to restrict the frequencies in our data to *f* < .1 Hz [47]. Next, we extracted the mean time series from two regions (deep temporal WM and bilateral lateral ventricles) in the post-ICA filtered data. The goal of including these two nuisance regressors was to remove residual cardiorespiratory physiological noise that would be captured by signal changes in the WM and ventricles [48–51] and was not removed by the ICA cleanup. The two nuisance regressors (timeseries from WM and ventricles) were regressed out using the general linear model with FEAT 6.00 (FMRI Expert Analysis Tool; http://www.fmrib.ox.ac.uk/analysis/research/feat/). Finally, we calculated the standard deviation (SD_BOLD_) across the whole timeseries for each voxel and smoothed the images with 6mm Gaussian kernel. We smooth the data as the last step to preserve the original localization of the signal throughout all the preprocessing steps. The resulting SD_BOLD_ maps were upsampled to MNI space using registration steps described above. To restrict all multivariate analyses to the GM, we masked the SD_BOLD_ maps with the GM tissue prior provided in FSL, thresholded at probability > 0.37, as described earlier in [26]. The SD_BOLD_ maps of the 100 participants are deposited at http://neurovault.org/collections/608/.

### PLS multivariate analysis of relations among SD_BOLD_, PA and CRF

The behavioral PLS analysis [52,53] began with a correlation matrix (CORR) between our variables of interest (PA and CRF) and each voxel’s signal (SD_BOLD_); correlations were calculated across subjects. Then, this CORR matrix was decomposed via singular value decomposition (SVD): SVD_CORR_ = *USV’*. This decomposition produces a left singular vector of behavioral weights (*U*), a right singular vector of SD_BOLD_ weights (*V*), and a diagonal matrix of singular values (*S*). In other words, this analysis produces orthogonal latent variables (LVs) that optimally represent relations between PA and CRF measures and SD_BOLD_ in GM voxels. Each LV contains a spatial pattern depicting the brain regions where the SD_BOLD_ shows the strongest relation to PA and CRF. Each brain weight (in *V*) is proportional to the correlation between PA and CRF with SD_BOLD_. To obtain a summary measure of each participant’s expression of a particular LV pattern, we calculated within-person “brain scores” by multiplying each voxel (*i*)’s weight (*V)* from the particular LV (*j*) produced from the SVD in equation (1) by the SD_BOLD_ value in that voxel for person (*k*), and summing over all (*n*) brain voxels: $\sum_{i\;=\;1}^{n}V_{ij}{SD\;}_{ik}$. Thus, in a single measure, a brain score indicates the degree to which a subject expresses the multivariate spatial pattern captured by the particular LV. Significance of detected relations between multivariate spatial patterns of SD_BOLD_, PA and CRF was assessed using 1000 permutation tests of the singular value corresponding to each LV. A subsequent bootstrapping procedure revealed the robustness of voxel saliences across 1000 bootstrapped resamples of our data [54]. By dividing each voxel’s mean salience by its bootstrapped standard error, we obtained “bootstrap ratios” as normalized estimates of robustness. We thresholded bootstrap ratios at a value of ≥ 3.00, which approximates a 99% confidence interval and corresponds to p < .001.

### DTI analysis

Visual checks were performed on every volume of the raw data of every participant by AZB. Seventy-four participants had good quality DTI data. In one dataset, one volume with the corresponding b-vectors and b-values was deleted from the dataset before processing due to an artifact. Next, DTI data were processed using the FSL Diffusion Toolbox v.3.0 (FDT: http://www.fmrib.ox.ac.uk/fsl) in a standard multistep procedure, including: (a) motion and eddy current correction of the images and corresponding b-vectors; (b) removal of the skull and non-brain tissue using the Brain Extraction Tool [41]; and (c) voxel-by-voxel calculation of the diffusion tensors. Using the diffusion tensor information, FA maps were computed using DTIFit within the FDT. All motion- and eddy-current outputs, as well as FA images were visually inspected.

We used TBSS [55,56], a toolbox within FSL v5.0.1, to create a representation of main WM tracts common to all subjects (WM “skeleton”). This included: (a) nonlinear alignment of each participant’s FA volume to the 1 x 1 x 1 mm^3^ standard Montreal Neurological Institute (MNI152) space via the FMRIB58_FA template using the FMRIB’s Nonlinear Registration Tool (FNIRT, [57]; http://www.doc.ic.ac.uk/~dr/software); (b) calculation of the mean of all aligned FA images; (c) creation of the WM “skeleton” by perpendicular non-maximum-suppression of the mean FA image and setting the FA threshold to 0.25; and (d) perpendicular projection of the highest FA value (local center of the tract) onto the skeleton, separately for each subject. The outputs of all the above processing steps were carefully inspected by AZB. To obtain a global FA measure, we averaged FA over the whole skeleton for each participant.

### Statistical analyses

All statistical analyses were performed using SPSS (v.16, SPSS Inc., Chicago, IL, USA). We used multiple step-wise hierarchical linear regressions (with chronological age and gender) to investigate the relationships between brain scores expressing the CRF/PA—SD_BOLD_ relationship and motion parameters. The brain scores of the PLS model relating SD_BOLD_ to PA and CRF were above 2.5 SD for two participants, and were Winsorized before regression analyses and creating scatterplots. The relative motion was ln-transformed for correlations as it was positively skewed.

The demographic data, FA, CRF and PA values, and brain scores are available in (S1 Dataset).

## Results

### Correlations between CRF, PA, and SD_BOLD_ (CRF/PA—SD_BOLD_ model)

**Fig 1:**
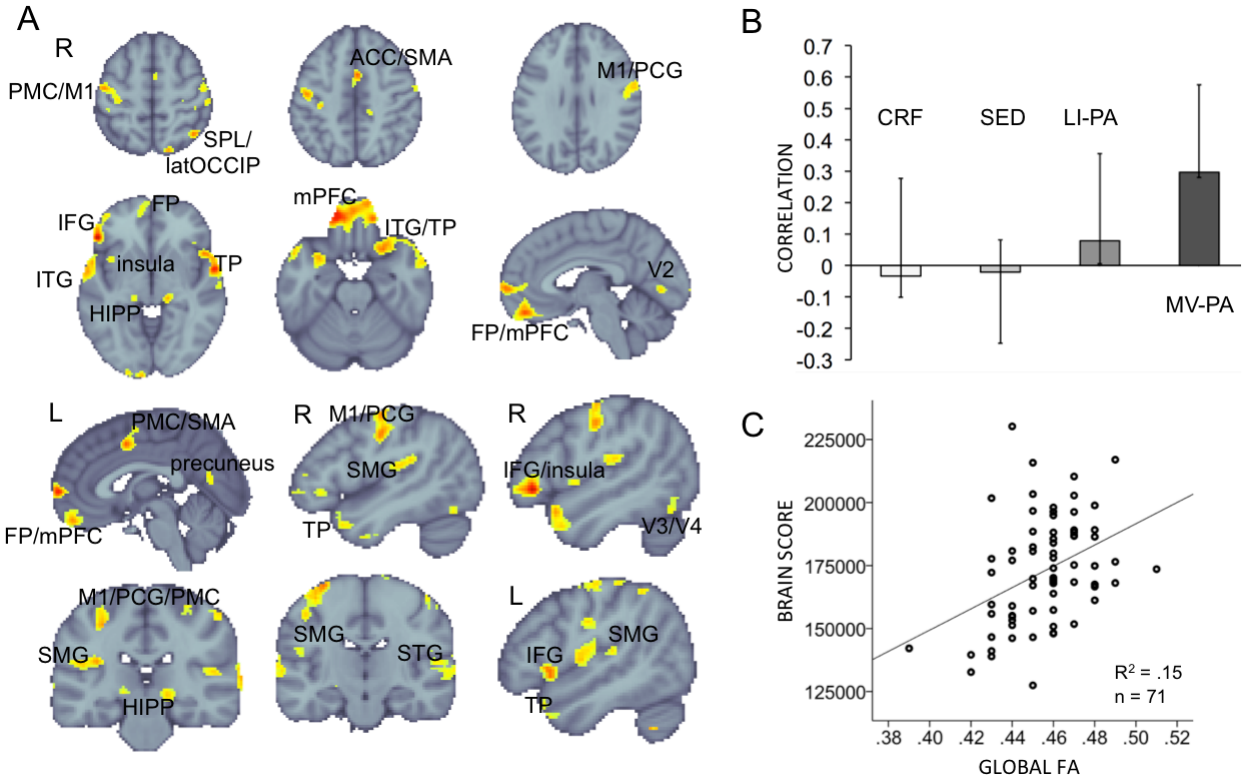
Multivariate relationships between CRF, PA, and SD_BOLD_ (the CRF/PA—SD_BOLD_ model). **A:** PLS spatial pattern of the CRF/PA—SD_BOLD_ model. Red-yellow regions indicate greater SD_BOLD_ with greater LI-PA and MV-PA. Significant regions: bootstrap ratio ≥3.00. Abbreviations as in Table 2. **B:** Correlation magnitudes (Pearson r) between CRF, sedentary time, LI-PA, MV-PA, and SD_BOLD_ during rest (permuted p<0.001, error bars represent bootstrapped 95% confidence intervals). CRF and sedentary time did not contribute to the LV as their error bars cross zero.

To identify multivariate patterns of relations between CRF, the three PA measures, and SD_BOLD_ in the entire GM we performed behavioral PLS analysis. We refer to it as the CRF/PA—SD_BOLD_ model. The behavioral PLS analysis yields orthogonal latent variables (LVs) that optimally represent relations of SD_BOLD_ in GM voxels with CRF and number of hours spent on PA at three intensities. The analysis yielded one significant LV (permuted p = 0.040, 52.63% cross-block covariance explained by this LV), suggesting that more LI-PA and MV-PA was related to greater SD_BOLD_ in multiple GM regions (Fig 1A and 1B). CRF and sedentary time did not significantly contribute to the model. Peak voxels’ location and bootstrap ratios for the CRF/PA—SD_BOLD_ model are reported in Table 2.

**Table 2:** Significant clusters representing the CRF/PA—SD_BOLD_ model from Fig 1.

Regions	MNI coordinates (mm)			BSR	Cluster size (voxels)
	x	y	z		
L IFG	-50.0	32.0	-4.0	4.9640	233
L FP/mPFC	-14.0	42.0	-22.0	4.8574	1496
L FP/SFG	2.0	64.0	2.0	4.6585	318
R TP	36.0	22.0	-32.0	4.6298	720
R STG/TP	60.0	0.0	-4.0	4.6090	1332
L pOPER/SMG	-40.0	-32.0	20.0	4.3541	258
L STG	-62.0	-10.0	8.0	4.3281	846
R latOCCIP/IPC	16.0	-78.0	54.0	4.2861	58
R PCG/M1	62.0	0.0	24.0	4.2823	340
R SPL/latOCCIP	40.0	-60.0	58.0	4.2524	49
R ACC/SMA	2.0	6.0	44.0	4.2336	147
L PCG/M1	-48.0	-12.0	42.0	4.1418	585
L PCG/SPL	-30.0	-40.0	68.0	4.1398	81
L V3/V4	-40.0	-90.0	4.0	4.0959	57
L V1	-8.0	-102.0	0.0	4.0007	119
L TP	-30.0	8.0	-24.0	3.8401	168
R cerebellum	50.0	-52.0	-42.0	3.8265	12
R FP	8.0	70.0	16.0	3.8245	20
R HIPP	16.0	-28.0	-4.0	3.7913	75
R PCG/SMG/SPL	52.0	-30.0	58.0	3.7820	109
L latOCCIP/ITG	-54.0	-66.0	-10.0	3.6922	72
R precuneus/latOCCIP	18.0	-76.0	38.0	3.6450	26
L V2	-6.0	-78.0	0.0	3.6431	45
L insula	-38.0	10.0	-6.0	3.5591	36
L TP	-30.0	20.0	-34.0	3.5372	45
R SMG/SPL	50.0	-46.0	52.0	3.4958	14
L FP	-26.0	66.0	14.0	3.4956	46
R precuneus	2.0	-66.0	18.0	3.4385	40
R PCG/PCC/precuneus	14.0	-32.0	44.0	3.3899	30
L HIPP	-16.0	-28.0	-4.0	3.3413	19
L ITG	-46.0	0.0	-40.0	3.2206	15
L FP/mPFC	-8.0	62.0	-18.0	3.1092	16

All peaks and clusters were determined using a voxel extent ≥10, minimum distance 10mm, and bootstrap ratio (BSR) ≥3.00. MNI, Montreal Neurological Institute (mm). R: right; L: left; IFG: inferior frontal gyrus, FP: frontal pole; mPFC: medial prefrontal cortex, SFG: superior frontal gyrus; TP: temporal pole; STG: superior temporal gyrus; pOPER: parietal operculum; SMG: supramarginal gyrus, lat: lateral; OCCIP: occipital cortex; IPC: intraparietal cortex; PCG: post central gyrus; M1: primary motor cortex; SPL: superior parietal lobule; ACC: anterior cingulate cortex; SMA: supplementary motor area; V3/V4: visual cortex III, IV; V1: primary visual cortex; HIPP: hippocampus; ITG: inferior temporal gyrus; V2: secondary visual area; PPC: posterior parietal cortex.

### CRF/PA—SD_BOLD_ model: relationship to structural brain connectivity

In our previous studies we showed that behaviorally relevant SD_BOLD_ is related to whole-brain WM microstructure in older adults [21] and that greater PA was positively associated with WM microstructure [12]. Therefore, after showing that greater LI-PA and MV-PA is beneficial for brain function (i.e. related to greater SD_BOLD_), we tested the hypothesis that the CRF/PA- related brain pattern of SD_BOLD_ would be associated with WM microstructure. We expected that participants who engage in more LI-PA and MV-PA should show an advantage in both brain structure and function, and therefore greater WM microstructure should be related to greater “brain scores” from the CRF/PA—SD_BOLD_ model. “Brain score” is a summary measure of each participant’s expression of the significant LV pattern from Fig 1. Thus, a person with a higher brain score showed higher LI-PA and MV-PA and greater SD_BOLD_ in the voxels depicted in Fig 1A. As there was a negative association between FA and age, and brain scores of CRF/PA—SD_BOLD_ model and age (r = -.23 p = .021, n = 100), we performed a hierarchical multiple linear regression analysis to investigate age-independent links between the brains scores and WM microstructure. The brain scores were the dependent variable, age was the first independent variable and global FA was the second independent variable. We found that higher FA accounted for a significant amount of variance in the CRF/PA—SD_BOLD_ association, in addition to variance related to age (R^2^ change_age_ = 0.034, F change_age_ = 2.41, df = 69/1, p-value = .125; R^2^ change_globalFA_ = 0.121. F change_globalFA_ = 9.753, df = 68/1, p-value = .003). We note that there was a trend toward a positive relationship between MV-PA and FA (r = .20 p = .101, n = 71), while CRF, sedentary time, and LI-PA were not associated with global FA.

### Exploring the effects of in-scanner motion on CRF/PA—SD_BOLD_ associations

We found that people who engage more in LI-PA and MV-PA show greater SD_BOLD_ (CRF/PA—SD_BOLD_ model, Fig 1). If our findings were driven by individual differences in motion then this would predict that more active people moved more in the scanner. This is unlikely but we tested this to ensure that our results were not confounded by motion. We performed three analyses: 1) We tested the in-scanner motion—SD_BOLD_ relationship directly in a behavioral PLS model with motion parameters from the resting state acquisition as the behavioral variable; 2) We related CRF and PA measures to in-scanner motion; 3) We correlated the brain scores from the CRF/PA—SD_BOLD_ model with motion parameters.

The relative motion (volume-to-volume displacement of subject’s head) should have the most influence on moment-to-moment variability in the BOLD signal during the resting state acquisition. Therefore, we used the mean relative motion quantified by the motion correction algorithm during preprocessing as the measure of the average volume-to-volume displacement of subject’s head during the scan. If motion during scanning contributed to the CRF/PA—SD_BOLD_ relationship, we expected: 1) a significant positive relationship between mean relative motion and SD_BOLD_; 2) a significant positive relationship between mean relative motion and PA; and 3) a significant positive relationship between mean relative motion and the brain scores from the CRF/PA—SD_BOLD_ model.

Our results did not support the possibility that in-scanner motion contributes to the SD_BOLD_ and the CRF/PA—SD_BOLD_ associations. First, the PLS behavioral analysis relating relative motion to SD_BOLD_ relationship did not yield a significant LV (p = .375), suggesting that there was no relationship between moment-to-moment subjects’ motion in the scanner and the moment-to-moment variability in the BOLD signal. Second, we found that participants with higher CRF, PA, and less time spent in sedentary behavior showed not more, but less relative motion during the resting state scan (CRF: r = -.38, p < .001, n = 100; sedentary time: r = .23, p = .022, n = 100; MV-PA: r = -.27, p = .008, n = 100). LI-PA was not related to relative motion. Third, we found that relative motion was not related to the brain scores representing the CRF/PA—SD_BOLD_ model (r = .005, p = .957, n = 100).

## Discussion

We used a whole-brain, multivariate approach to investigate the associations of objective indices of physical fitness and activity (CRF and PA) with brain functional resting state properties (moment-to-moment variability in the BOLD signal) in healthy older adults. This allowed us to demonstrate that older adults who engage in more PA (LI-PA and—to a greater extent—MV-PA) have greater SD_BOLD_ in multiple regions, while CRF and sedentary behavior did not significantly contribute to variance in SD_BOLD_. Next, the inter-individual differences in the PA—SD_BOLD_ relationships were associated with global WM microstructure, above and beyond the effects of chronological age. Moreover, we demonstrated that the in-scanner motion did not drive the association of PA with SD_BOLD_. Here we discuss the implications of this first demonstration of an association between objectively measured PA, brain function, and structure in an older population.

### SD_BOLD_ as a candidate functional correlate of brain health

In the current study, we showed that older, low-active adults who engage regularly in more LI-PA and MV-PA have greater variability in the spontaneous low-frequency BOLD signal. Given that previous evidence has shown that: 1) greater SD_BOLD_ in older age relates to better cognitive functioning; 2) the brain’s network organization may benefit from greater SD_BOLD_ in specific regions; and that 3) regions showing the PA—SD_BOLD_ relationship overlap with regions where some conventional neural functional measures are related with CRF and PA, we interpret our findings to suggest that a positive relationship between SD_BOLD_ and PA reflects better functional brain health.

First, recent studies exploring the significance of SD_BOLD_ for cognitive performance in aging demonstrated that older adults show lower SD_BOLD_ than young adults, and that greater SD_BOLD_ in multiple GM regions is related to faster and more consistent performance on a perceptual matching task [20], and better fluid abilities and memory in older adults [21]. These previous findings suggest that preserving optimal (i.e. higher) levels of SD_BOLD_ in specific brain regions in advanced age is beneficial for cognitive functioning. Here we show that SD_BOLD_ may serve as one of the correlates of functional brain health related to physical activity and health.

Second, many of the brain regions showing the PA- SD_BOLD_ relationship play an important role in brain network organization. Therefore, our results support the notion that the brain network function should benefit from their greater SD_BOLD_ [21], assuming it represents greater dynamic range or kinetic energy of neural processing [18]. Specifically, regions identified in the current study play an important role in major resting state networks: M1/PMC constitute the hubs of the motor network [58], precuneus, mPFC, HIPP, medial STG, SPL, lateral OCCIP are hubs of the default mode network (DMN; [59,60]), and FP, anterior insula, SMG, and ACC constitute networks that have been associated with executive control and salience detection [61]. These hub regions have been defined in structural and functional network analyses as highly connected within a certain network or community (DMN, motor [62–65]) or support communication between distinct networks (63–65). Therefore, we speculate that greater SD_BOLD_ in these hub regions likely reflects neural processing related to integrating local and distributed neural communities. In addition, several regions identified in the current study (e.g. precuneus, insula, ACC, SMG) overlap with regions where greater SD_BOLD_ is related to cognitive performance (21), supporting the claim that cognitive performance is thought to depend on modulating and combining the activity of neural networks in different ways [66,67].

Third, the observed relationship between PA and SD_BOLD_ is in line with previous findings linking physical health, measured as CRF, with brain function, especially in regions showing PA- SD_BOLD_ relationship in the current study. For example, older [13] or middle-aged adults [14] with greater CRF have greater BOLD signal amplitude in fronto-parietal regions during a selective attention task [13] or in hippocampus, precuneus, insula, cingulate, and other frontal, temporal, and occipital regions during successful spatial encoding [14]. Importantly, there is growing evidence from randomized control trials in older adults that 12-month bi-weekly resistance training can increase BOLD response during a selective attention task in the anterior temporal cortex and insula [25], a 6-month aerobic training program can enhance fronto-parietal [13] or medial prefrontal BOLD response [14], and a 12-month aerobic walking program increases functional connectivity between regions in the default and frontal executive network [16]. In addition, endurance-trained middle-aged adults (age 40–65) have greater working memory-related neural activation in multiple temporal, frontal, and parietal regions than their sedentary peers [15]. Together, these studies suggest that fitter and more active adults show greater BOLD signal amplitude or greater functional connectivity during task performance. We speculate that the mechanisms of greater amplitude of BOLD signal fluctuations could be similar to greater SD_BOLD_ observed in more physically active older adults in our sample. We plan to investigate this claim directly in future studies linking SD_BOLD_ with functional connectivity and BOLD amplitude. In addition, further investigations (such as time frequency analysis, perfusion imaging, calibrated fMRI, and optical imaging) are needed to tease apart the effect of neural, vascular, and neurovascular coupling aspects of BOLD signal on SD_BOLD_ in relation to CRF and PA. In sum, SD_BOLD_ is a candidate brain health index that could unify and provide a better explanation of previous mixed findings based on different conventional functional neuroimaging measures.

### CRF and PA are not equivalent in their role in functional brain health

We did not observe the expected positive relationship between CRF and SD_BOLD_. We suggest that some aspects of lifestyle behaviors that influence brain function and aging may not be captured by CRF. In addition, a genetic component of CRF [68] that adds to greater CRF regardless of PA may contribute to the associations between CRF and brain function. Alternatively, motivation or subjective threshold of exhaustion may influence the value of VO_2_max reached during the treadmill test, especially in older low active adults, and therefore add noise to the CRF score.

In addition, it is possible that with careful preprocessing of the resting state data we removed some variance in the BOLD signal of vascular origin that would be related to cardiovascular aspects of physical health captured by CRF. As noted earlier, additional investigations with different imaging and analytical approaches are needed to tease apart vascular from neural aspects of SD_BOLD_. We highlight that in the current study we demonstrated in three different analyses that the relationship between PA and SD_BOLD_ was not driven by the motion during resting state acquisition. Neither was total relative motion directly related to SD_BOLD_, nor was motion related to brain scores reflecting the PA—SD_BOLD_ relationship. Moreover, adults who had higher CRF and PA showed less motion during the resting state. We speculate this may be related to either greater physical comfort of more fit/active people in staying still (or some physical discomfort that also prevents less active individuals from engaging in PA), or their better adherence to experimental instructions.

Finally, sedentary time did not significantly contribute to SD_BOLD_. We speculate that in our relatively low-active and low-fit sample the variance in sedentary time was not sufficient to detect robust relationships with brain function. In other words, it was LI-PA and MV-PA rather than sedentary behavior that made a difference with respect to brain functional properties in this low active, low fit but otherwise healthy older sample. An ongoing exercise intervention study including this group of participants will shed more light on the discrepancy between CRF, PA, and sedentariness in relation to brain function.

### Both WM structure and GM function benefit from PA

We showed that older adults with greater global FA exhibited more positive brain scores, i.e. had greater PA and greater SD_BOLD_. Our study therefore further extends previous reports of a positive relationship between WM microstructure and physical fitness and activity, as measured by either CRF or PA [12,69–72]. We propose that anti-inflammatory and pro-myelination effects of PA on the aging brain may explain the link between PA-related SD_BOLD_ and WM microstructure. For example, increased PA may protect the oligodendrocytes against damage, such as related to oxidative stress [73,74] and the age-related reduction in myelin integrity [75]. In addition, PA and exercise reduce systemic markers of inflammation [76], while increased inflammatory markers are associated with lower WM microstructure in healthy non-demented older adults [77,78]. Together, our data suggests that PA benefits both WM microstructure and GM function, as patterns and magnitude of SD_BOLD_ at rest reflecting brain functional health are supported by WM microstructure in healthy aging. Future studies will assess the role of GM volume and density in these relationships.

## Conclusions

We demonstrated a positive association between PA, variability in the BOLD signal, and WM microstructure in healthy low-active older adults. We found that greater LI-PA and MV-PA coincided with greater SD_BOLD_ in regions where function is known to benefit from greater CRF and PA, and which play an important role in within and between-network communication in the brain. Therefore, our results suggest that greater PA in older age and the related physiological and neural mechanisms may support optimal neural processing in key regions, as well as WM integrity of tracts connecting these distributed regions. We conclude that SD_BOLD_ is a promising neural correlate of functional brain health in healthy older adults and the objective assessment of PA is an important tool in investigating functional aspects of brain health. The ongoing longitudinal and intervention studies will shed more light on the potential of SD_BOLD_ in detecting changes in brain function as a result of increased PA, cognitive stimulation, and dietary supplements.

## Supporting Information

S1 Dataset Demographic, DTI, physical activity, fitness, and brain score data for the 100 participants. “Win” in the variable name indicates this variable was Winsorized. The **SD**_**BOLD**_ maps of the 100 participants are deposited at http://neurovault.org/collections/608/.
(XLSX)
